# Implementation of the World’s largest measles-rubella mass vaccination campaign in Bangladesh: a process evaluation

**DOI:** 10.1186/s12889-019-7176-4

**Published:** 2019-07-10

**Authors:** Haribondhu Sarma, Ashwin Budden, Sharmin Khan Luies, Stephen S. Lim, Md. Shamsuzzaman, Tahmina Sultana, Julie K. Rajaratnam, Laura Craw, Cathy Banwell, Md. Wazed Ali, Md. Jasim Uddin

**Affiliations:** 1 International Centre for Diarrhoeal Disease Research, Bangladesh (icddr,b) 68 Shahid Tajuddin Ahmed Sarani, Dhaka, 1212 Bangladesh; 2 Research School of Population Health, the Australian National University, Acton, ACT 2601 Australia; 3 PATH, 2201 Westlake Avenue, Suite 200, Seattle, WA 98121 USA; 4 Institute for Health Metrics and Evaluation, 2301 5th Ave Suite 600, Seattle, WA 98121 USA; 5 Gavi, the Vaccine Alliance, 2 Chemin des Mines, Geneva, 1202 Canton de Genève, Switzerland

**Keywords:** Measles and rubella, Mass vaccination campaign, Process evaluation, Bangladesh, ‘Gavi’ and ‘immunization’

## Abstract

**Background:**

Gavi, the Vaccine Alliance, supported a mass vaccination Measles-Rubella Campaign (MRC) in Bangladesh during January–February 2014.

**Methods:**

We conducted a mixed-method process evaluation to understand the successes and challenges in implementation of the MRC. We reviewed documents for the MRC and the immunization programme in Bangladesh; observed meetings, vaccination sessions, and health facilities; and conducted 58 key informant interviews, 574 exit interviews with caregivers and 156 brief surveys with stakeholders involved in immunization. Our theory of Change for vaccination delivery guided our assessment of ideal implementation milestones and indicators to compare with the actual implementation processes.

**Results:**

We identified challenges relating to country-wide political unrest, administrative and budgetary delays, shortage of transportation, problems in registration of target populations, and fears about safety of the vaccine. Despite these issues, a number of elements contributed to the successful launch of the MRC. These included: the comprehensive design of the campaign; strong partnerships between immunization authorities in the government system, Alliance partners, and civil society actors; and motivated and skilled health workers at different levels of the health system.

**Conclusions:**

The successful implementation of the MRC in spite of numerous contextual and operational challenges demonstrated the adaptive capacity of the national immunization programme and its partners that has positive implications for future introductions of Gavi-supported vaccines.

## Background

Mass vaccination campaigns are important mechanisms to control and eliminate infectious diseases in low- and middle-income countries (LMICs). In recent years, measles-rubella campaigns (MRCs) have helped countries achieve their national immunization coverage targets [1–9]. Global measles control programs have increasingly used campaigns to supplement routine immunization and reduce measles-related morbidity [10]. However, measles and rubella continue to cause significant morbidity and mortality in children, despite the availability of low-cost vaccines for over 40 years [11].

Prior evaluations of MRCs in different countries have estimated vaccine coverage [2, 7], described immunization settings, and waste disposal procedures [5], assessed the quality of vaccine [12], and documented campaign outputs [4]. They called attention to a number of areas for improvement in campaign operations, including the need for pre-campaign meetings to foster strong coordination between national and district levels; timely training, workshops, and post-campaign review; planning for adequate logistics; micro-planning for sub-district and district levels; and types and duration of vaccination sessions [1, 13]. Some studies also suggested that successful implementation of MRCs can be hampered by systemic challenges that include vaccine availability, political unrest, and inadequate training for health workers [2, 3, 13].

To reduce the measles and rubella disease burden, the Expanded Program on Immunization (EPI) in Bangladesh, managed by the Ministry of Health and Family Welfare (MoHFW), conducted an MRC from 25 January to 13 February 2014. The national campaign targeted more than 52 million children aged 9 months to 14 years. Gavi, the Vaccine Alliance (commonly known as Gavi) funded the government to conduct the campaign, which included planning, training the health workforce, mobilizing demand in communities, and procuring the MR vaccine. Gavi also supported a Full Country Evaluation (FCE) in Bangladesh, from 2013 to 2016, to understand and measure vaccine coverage, barriers to, and drivers of, improvement of the immunization program, with an emphasis on its contribution of Gavi [14]. The FCE team conducted a coverage survey in a high performing and a low performing division in Bangladesh to measure coverage of Measles-Rubella (MR) vaccination before and after the MRC [15]. The results of that survey showed significant improvement of MR vaccination coverage in both the areas. MR vaccination coverage in high performing division was 4% before MRC and it increased to 95% after MRC. In the low-performing division, MR coverage increased from 11 to 85% after MRC [15].

The results of the outcome survey demonstrated the effectiveness of the MRC. In general, however, outcome evaluations do not shed light on why and how programs achieved such successful outcomes, or provide information that would aid replication or scale-up of the program in other settings. Process evaluations can fill this knowledge gap by assessing whether program activities have been implemented as intended [16], and by highlighting challenges and success and the process that contributed to them. As part of the FCE, we conducted a process evaluation of the MRC. It is expected that this evaluation will provide information for improving the operations of the immunization programme in Bangladesh, for ensuring accountability at the country level to inform the implementation of future vaccination campaign.

## Methods

**Figure 1:**
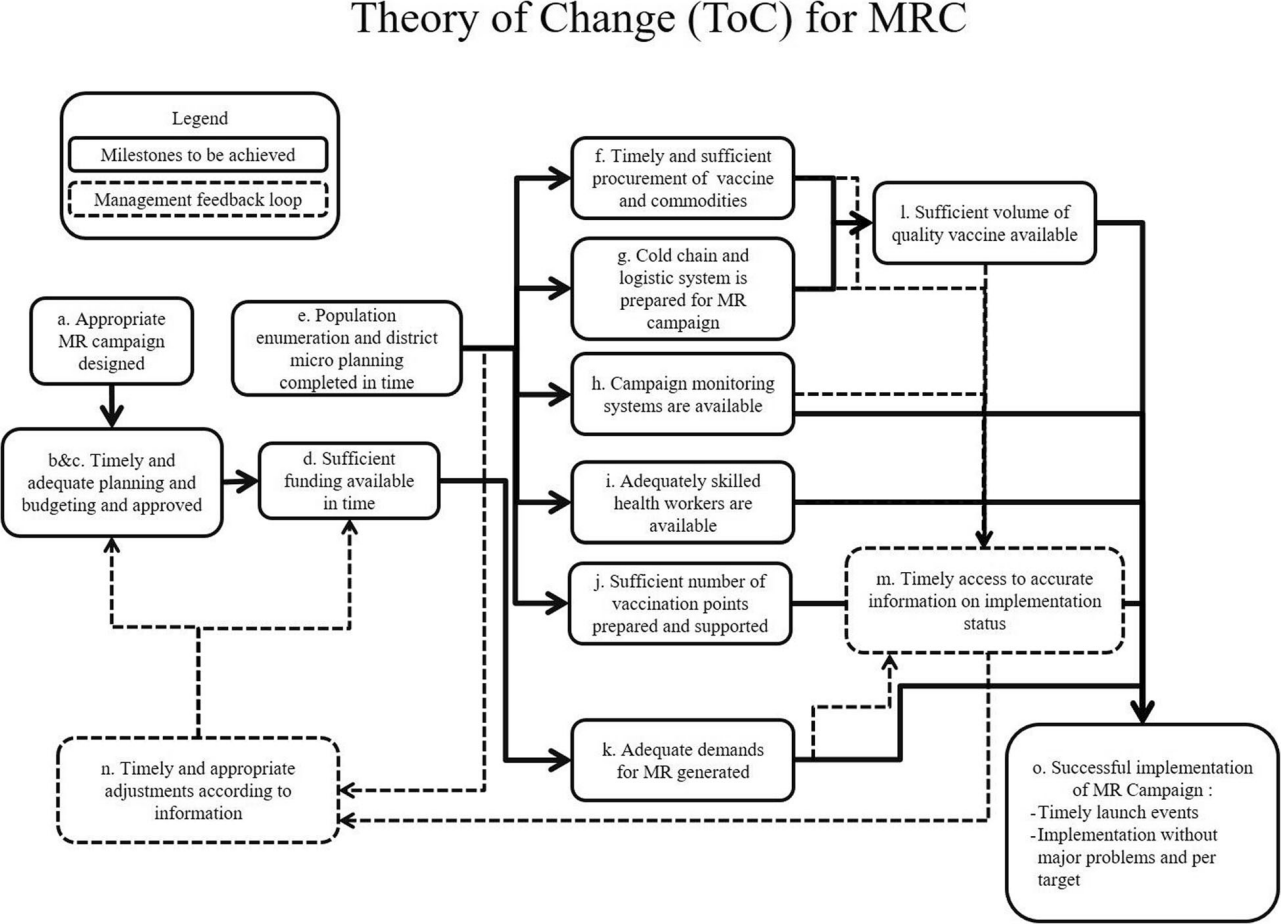
Theory of Change (ToC) for MRC

The process evaluation was one of the aspects of a multi-faceted evaluation of the MRC in Bangladesh. It employed a cross-sectional, retrospective and mixed methods study design, covering the full results from inputs to impact, to examine the planning and implementation phases of the MRC. The evaluation framework entailed inter-related steps:

- Development of a theory of change which provided an analytical framework for defining ideal program implementation milestones and indicators to compare with the actual processes [17, 18]. Our theory of change organized the process around a series of high-level milestones which, ideally, must be accomplished for the output to be achieved (Fig. 1). For each milestone of the Theory of Change, we developed indicators to reflect the fidelity of the implementation, (i.e. whether the program was delivered as intended).
- Process tracking included a document review (*n* = 31), observations of immunization stakeholders’ meetings (*n* = 4) and vaccination sessions (*n* = 144), and facility assessments (*n* = 200). Process tracking, which was the primary means of monitoring the progress of the MRC implementation activities, compared the actual processes with the ideal processes defined in the Theory of Change. The document review captured a wide range of written sources pertaining to all phases of Gavi support. We obtained documents through direct requests to the stakeholders; through access to routine distribution channels, such as email list and web sites; and through database searches. The documents included country expressions of interest and applications to Gavi for support; review- and decision-related correspondences from Gavi; MoHFW planning documents such as the operational plans of Maternal Neonatal Child and Adolescent Health, the national health sector plan (HPNSDP 2011–2016), health bulletins of the Directorate General of Health Services (DGHS), Comprehensive Multi-Year Plan; and meetings minutes of the interagency coordination committee and technical sub-committee.
- In-depth key informant interviews (*n* = 58) with purposively selected EPI stakeholders, including EPI managers at different levels of the health system and representatives from partner organizations and MoHFW; exit interviews with caregivers (*n* = 574) of beneficiary children; and a brief survey (*n* = 156) of immunization service providers.

The key informants were national-level stakeholders who were involved in policy-making; design, planning, and implementation of the campaign while immunization stakeholders were direct service providers at the community or facility level.

We also conducted root cause analysis [19] to understand and document how particular challenges and successes around the MRC implementation have occurred. These involved group exercises through collaborative brainstorming, critical assessment of evidence gathered (both qualitative and quantitative), and visual diagramming of causal chains (connecting observed challenges, successes and consequences to assumed root causes).

These methods were employed to identify and prioritize gaps in understanding key programmatic challenges and successes and to further investigate them and explain their underlying causes.

We implemented the process evaluation at the national, district, and community levels in both rural and urban areas of Bangladesh. We purposively selected two administrative divisions, out of seven in the country, based on EPI coverage data [20]: Rajshahi (high-performing), with 85.8% of children fully immunized and Sylhet (low-performing), with 74.8% of children fully immunized. The respective population-size (PS) of Rajshahi and Sylhet division at the time of the evaluation was 18,484,858 and 9,910,219 [21]. We then selected Joypurhat District (high-performing), with 88.4% fully vaccinated (PS = 950,441) from the Rajshahi division and Sylhet District, (low-performing), with 74.9% (PS = 3,434,188) from the Sylhet division. Similarly, we selected two City Corporations (Municipality Corporation that act as local governments): Rajshahi City Corporation (high-performing), with 88.8% fully vaccinated (PS = 449,756) and Sylhet City Corporation (low-performing), with 62.4% (PS = 531,663) [20, 21]. A detailed explanation of the selection of study sites has been reported elsewhere [15].

We collected quantitative data through direct observations of vaccination sessions, structured exit interviews, and provider surveys to estimate the proportion of respondents reporting on the study indicators, such as mothers’ perceptions on the campaign, their motivations, and experiences with the healthcare providers regarding the services they received from the campaign. We performed univariate analysis and equality of two proportions test (Fisher’s exact test/t- test) on these data and calculated 95% confident intervals (CI) for each estimate (percentage/mean). The confidence interval presented in the tables calculated considering cluster in account. After performing Fisher’s exact test for categorical variables and t-test for continuous variables, *p*-values were used to demonstrate the statistical differences between two areas (high-performing and low-performing).

Qualitative analysis commenced from the beginning of qualitative data collection so that we could already identify informational gaps and saturation points (meaning that little new information was being gathered on particular topics [22]). Our analysis followed Patton’s approach to qualitative evaluation [23], in which we started with data coding, then proceeded to data reduction, display, and synthesis and interpretation of data. We used Atlas-ti software (version 6.2) for managing and analysing qualitative data. We performed root cause analysis on priority issues and questions that emerged from the initial analysis to clarify information gaps and confirm the findings. As part of our root cause analysis, we created flowcharts to visually depict the causal chains linking observed challenges and successes in the implementation of the MRC to their underlying causes.

## Results

The MRC in Bangladesh was the largest immunization campaign in the world to date, reaching roughly a target of 52 million children aged 9 months to < 15 years. Despite a number of challenges, the campaign was implemented successfully due to some key enabling factors. Detailed findings on challenges and successes are described below under four main themes.

### Design and availability of funds for MRC

The MoHFW planned to implement the MRC in two-phases within a three-week campaign period. During the first phase it carried out vaccination activities in educational institutions and during the second phase at EPI facilities. The MoHFW took timely initiatives to develop and update the MRC implementation process, and updated stakeholders at the sub-national level through memos and letters. Nevertheless, there were noted gaps in planning and budgeting line items in the absence of prior consultation with sub-national stakeholders.

**Fig. 2:**
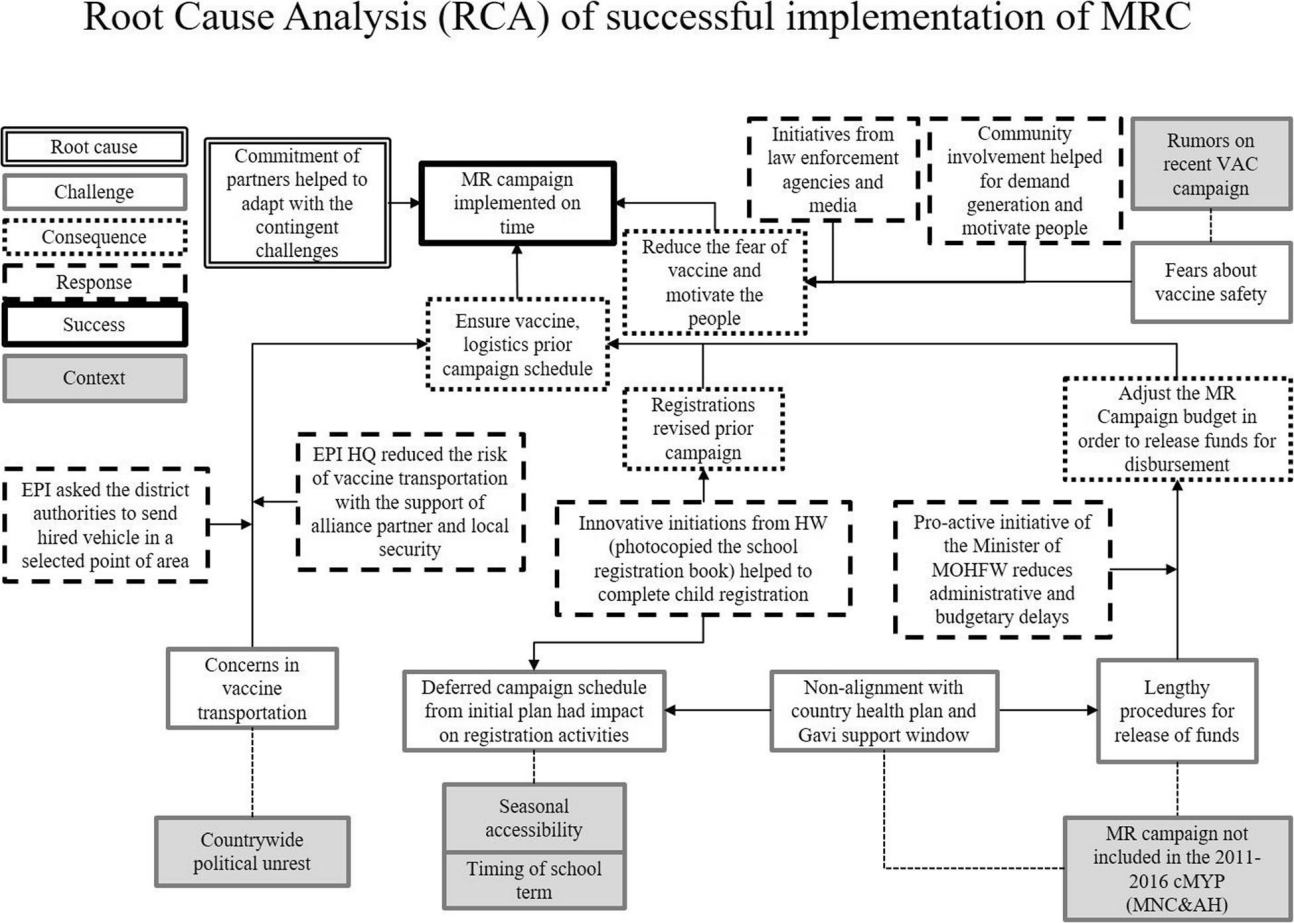
Root Cause Analysis (RCA) of successful implementation of MRC

Timely approval and allocation of the MRC funds depended on inclusion of the campaign budget in the Operation Plan of Maternal Neonatal Child and Adolescent Health, part of the comprehensive Multi-year Plan of MoHFW. The comprehensive Multi-year Plan had already included MR vaccination in the routine EPI schedule but not as a separate campaign activity. Hence, Gavi approved the application for support under the condition that the comprehensive Multi-year Plan and the respective Operation Plan would be eventually revised by including the MRC. However, without an updated comprehensive Multi-year Plan and Operation Plan, the budget for implementing the MRC could not be approved for the country, which led to a delay in start-up of the MRC. The Minister of the MoHFW attempted to reduce administrative delays by adjusting the MRC budget to release funds for disbursement at all administrative levels (Fig. 2). In response, the EPI rescheduled the MRC launch from November 2013 to January 2014.

Figure 2 shows the results of root cause analysis on the implementation of the MRC. Initially, there were a number of challenges that delayed the implementation of the MRC, for example, concern about safe and on-schedule vaccine transportation, with the root cause being identified as political unrest. Despite these challenges, the MRC was implemented in a timely and successfully manner due to the commitment of partners organizations such as United Nations Children’s Fund (UNICEF) and World Health Organization (WHO) that helped in mitigating to the contingent challenges, despite the political, seasonal and other contexts (Fig. 2).

Once the MoHFW received the decision letter from Gavi for funding the MRC, Gavi distributed the funds within the agreed timeframe mentioned in the decision letter for implementation of the MRC. The funds were also disbursed in a timely fashion to the district and sub-district levels. Almost all the service providers reported that they had received their training allowances. However, key informants at the sub-national level indicated that inadequate funds were allotted for volunteers’ refreshments, which discouraged them from participating in the MRC vaccination sessions. Concerned health workers spent their own money to ensure the participation of the volunteers in the sessions.

### Management of micro-planning, logistics, and cold chain for MR vaccine

The EPI reviewed and updated micro-plans prior to the campaign. Delayed distribution of micro-planning forms from national to the sub-national level created stress for health workers. However, health workers still performed micro-planning on time as they were motivated to do the extra work and were able to work beyond normal office hours; they also used their experiences from previous immunization campaigns (Fig. 2) to reflect on this campaign. One community health worker commented:

*Registration time was a constraint to our work efficiency. Allocating at least three months for registration activities is necessary for these types of program.*

Recurrent political unrest in country posed difficulties for distributing vaccines and supplies for the MRC. However, EPI had collected and stored buffer stock of vaccines at national, district, and sub-district levels. During outbreaks of unrest, the WHO provided vehicles to assist in distributing vaccines and other logistics to sites from the district to sub-district levels to ensure their availability (Fig. 2). Our observation of vaccination sessions revealed that the MR vaccine was available in about 99% (95% CI 97.0–100) of the sessions (Table 1). However, in all sessions we observed that there were inadequate quantities of soap, finger markers and cotton, which are necessary commodities for administering vaccines.

**Table 1:** Quality and adequacy of logistics, and quality of services provided during MR campaign

Parameter	High-performing division (Rajshahi) *N* = 72		Low-performing division (Sylhet) *N* = 72		*p-value	Total *N* = 144
Availability of logistic-materials in the vaccination sessions						
	%	95% CI	%	95% CI		% (95% CI)
Vaccine vial	98.6	97.0–100	100	–	0.314	99.3 (98.2–100)
Vaccine carrier	97.2	93.9–100	100	–	0.153	98.6 (96.4–100)
Diluents	100	–	100	–	–	100
AD syringe	100	–	100	–	–	100
Finger marker	62.5	44.1–80.9	47.2	40.1–54.4	0.065	54.9 (46.5–63.2)
Soap	13.9	0–28.1	4.2	0–8.6	0.043	9.0 (3.4–14.6)
Cotton	80.6	65.3–95.8	52.8	45.9–59.6	0.000	66.7 (52.7–80.7)
Quality and adequacy of logistics						
Fully-melted ice pack	0.0	–	1.4	1.1–1.7	0.314	0.7 (0–2.1)
Semi-frozen ice pack	34.7	23.7–45.7	73.6	63.4–83.8	0.000	54.2 (46.1–62.3)
Registration form	33.3	5.4–61.3	38.9	30.0–47.8	0.484	36.1 (21.3–50.9)
Adequate MR vaccine supplies	86.1	80.0–92.2	90.3	82.6–98.0	0.435	88.2 (82.9–93.5)
Adequate needles or syringes	90.3	78.7–100	94.4	90.5–98.4	0.355	92.4 (85.9–98.8)
Adequate both MR vaccine and needles or syringes	94.4	87.8–100	97.2	94.6–99.9	0.402	95.8 (91.9–99.8)
Duration of session (Mean ± SD)	4.3 ± 1.4 h		4.0 ± 1.2 h		0.170 4.1 ± 1.3 h	
Availability of firstline supervisor and volunteer						
Firstline supervisor	62.5	37.3–87.7	43.1	32.8–53.3	0.020	52.8 (40.1–65.5)
Volunteer	94.4	90.6–98.3	50.0	39.8–60.2	0.000	72.2 (56.1–88.4)
Quality of services provided to individual children	*N* = 462		*N* = 453		*p-value	*N* = 915
	%	95% CI	%	95% CI		% (95% CI)
Handwashing before vaccination	44.2	41.0–50.0	8.8	3.9–13.8	0.000	26.7 (16.0–37.3)
Top of the vaccine box /carrier snugly placed	90.9	86.0–95.8	77.7	68.8–86.6	0.000	84.4 (80.2–88.6)
Child’s finger marked after vaccination	94.7	92.4–97.0	70.1	60.5–79.6	0.000	82.4 (77.2–87.7)
Vaccinator put a tally after each vaccination	98.7	97.4–99.9	67.2	56.6–77.8	0.000	83.0 (77.8–88.1)
Information on adverse event/side-effects provided	5.1	2.9–7.2	6.2	4.4–8.1	0.471	5.6 (4.2–7.1)
Applied non-touch technique	100	–	99.6	99.1–99.9	0.174	99.8 (99.6–100)
Used AD syringes put into the safety box	99.1	98.5–99.7	95.8	91.1–100	0.000	97.5 (95.0–99.9)

* Fisher’s exact test

For the cold chain management of the MR vaccine, cold storage capacity and other logistic-materials were made available by EPI for the vaccine at the district and sub-district levels. The EPI used other public-sector facilities to mitigate challenges regarding insufficient vaccine storage facilities at the national level. In the meantime, managers at the district and sub-district levels had collaborated with other local-level stakeholders, including the local electricity department, ice-cream factories, and local government authorities to ensure adequate amount of ice packs were available for vaccine carriers. The EPI used freeze-tags to monitor the temperature of ice-lined refrigerators, which were used for storing large amounts of vaccines at the district and sub-district levels. In this campaign, the EPI used vaccine vial monitors to ensure the quality of the vaccine. However, during our observations of vaccination session we noted that only about 54% (95% CI 46.1–62.3) of the facilities used the appropriate ice packs (semi-frozen); MR vaccine vials and carriers were available in all the facilities, except in the urban areas of Rajshahi (Table 1).

### Availability of workforce and adaptability with a heavy workload

A total of 67,900 vaccinators and 241,000 volunteers were required to implement the MRC. The EPI ensured the availability of an adequate workforce by involving the private-sector medical colleges, nursing colleges, and local health institutions. The MRC involved a wide variety of roles, as noted by a national-level official stated:

*It’s true that some of the posts of health workers (Health Assistants) are vacant. Therefore, a large workforce was needed for the MR campaign, and they met the need for the required workforce by utilizing multi-sector staff, such as Health Assistants, Family Welfare Assistants, Health Inspectors, Family Planning Inspectors, Sub-Assistant Community Medical Officers, Medical Assistants, Family Welfare Visitors, and Sanitary inspectors.*

To ensure an adequate skill level among service providers for the MRC, the EPI developed a cascading training curriculum with 84% (95% CI 77.1–88.9) of the service providers receiving training for 2 days and 3.3% of the service providers receiving 3 days of training in one division (Table 2). However, training was not held in all districts and sub-districts as planned and the duration of training was curtailed a number of times due to political unrest.

**Table 2:** Perception of service providers and satisfaction of caregivers regarding MR campaign

Parameter	High-performing division (Rajshahi)		Low-performing division (Sylhet)		*p-value	Total
Duration of training	*N* = 60		*N* = 95			*N* = 155
	%	95% CI	%	95% CI		% (95% CI)
One day	0.0	–	24.2	16.5–34.0	0.000*	14.8 (10.0–21.4)
Two days	96.7	87.2–99.2	75.8	66.0–83.5		83.9 (77.1–88.9)
Three days	3.3	0.8–12.8	0.0	–		1.3 (0.3–5.1)
Perception of provider about adequacy of training						
Adequate	58.3	45.2–70.4	53.7	43.5–63.6	0.525	55.5 (47.5–63.2)
Reasons why training was inadequate	*N* = 25		*N* = 44		p-value	*N* = 69
	%	95% CI	%	95% CI		% (95% CI)
Duration of training was short	100	–	100	–	–	100
Training materials/logistics were insufficient	16.0	5.7–37.5	2.3	0.3–15.5	0.035	7.3 (3.0–16.6)
Training methods/techniques were not good	4.0	0.5–26.3	2.3	0.3–15.5	0.687	2.9 (−1.1–6.9)
Trainers were not good	0.0	–	15.9	7.5–30.5	0.035	10.1 (4.8–20.1)
Organizing campaign without hampering routine work	N = 60		*N* = 96		p-value	N = 156
	%	95% CI	%	95% CI		% (95% CI)
Very successful	75.0	62.2–84.6	50.0	40.0–60.0	0.003*	59.6 (51.7–67.1)
Somewhat successful	23.3	14.1–36.0	47.9	38.0–58.0		38.5 (31.1–46.4)
Not successful	1.7	0.2–11.5	2.1	0.5–8.1		1.9 (0.6–5.9)
Caregivers’ perception regarding benefits of MR campaign	*N* = 289		*N* = 285		p-value	N = 574
	%	95% CI	%	95% CI		% (95% CI)
Able to vaccinate child/children	77.50	72.3–82.0	78.3	73.0–82.7	0.817	77.9 (74.3–81.1)
Massive publicity motivates for vaccination	4.2	2.4–7.2	2.8	1.4–5.5	0.362	3.5 (2.3–5.3)
Aware about measles and rubella diseases	21.8	17.4–27.0	44.6	38.9–50.4	0.000	33.1 (29.4–37.1)
Reduces the fear of vaccination	2.8	1.4–5.5	3.2	1.6–6.0	0.779	3.0 (1.8–4.7)
Increases interest towards other vaccines	5.9	3.7–9.3	3.1	1.6–6.0	0.692	4.5 (3.1–6.6)
Changes attitude to go to the health centers for healthcare	6.6	3.7–9.5	0.7	0.2–2.8	0.000	3.7 (2.4–5.5)
Other	11.4	8.2–15.7	12.3	8.9–16.7	0.739	11.9 (9.4–14.8)
Decline to respond	2.4	1.2–5.0	2.8	1.4–5.5	0.170	2.6 (1.6–4.3)
Satisfaction of caregivers regarding MRC	*N* = 289		N = 285		*p*-value	N = 574
	%	95% CI	%	95% CI		% (95% CI)
Unsatisfied	0.4	0–2.4	0.7	0.2–2.8	0.011*	0.5 (0.2–1.6)
Satisfied	76.8	71.6–81.3	85.3	80..6–88.9		81..0 (77.6–84.0)
Very satisfied	22.8	18.3–28.1	14.0	10.4–18.6		18.5 (15.5–21.9)

* Fisher’s exact test

Some challenges were also observed in the quality of implementation of the MRC as service providers, in some places, did not practice standard vaccination procedures, such as using soap every time, marking the finger of the vaccinated child, tallying vaccinated children and informing caregivers about side-effects of the vaccine (Table 1). Additionally, some service providers had a heavy workload, working up to 3 hrs more than the normal working hours per day. Our observations of vaccination sessions revealed that 6 persons, including volunteers who managed the crowds, were required on average, to vaccinate about 208 children (Table 3). In our interviews, service providers reported that they had acquired useful skills from their involvement in previous immunization campaigns that they used to manage the MR campaign-related workload.

**Table 3:** Average number of children vaccinated and average number of vaccinators available in each session

Parameter	High performing division (Rajshahi) (%)	Low performing division (Sylhet) (%)	-**p-value	Total (*N* = 144) Average ± SD (95% CI)
	Average ± SD	Average ± SD		
Number of children vaccinated	175.0 ± 171.8	241.9 ± 309.8	0.112	208.4 ± 251.9 (166.9–249.9)
Number of vaccinators available	7.1 ± 3.8	4.2 ± 2.1	0.000	5.7 ± 3.4 (5.1–6.2)

** T-test

### Multiple partner involvement in developing advocacy strategy and IEC materials for the campaign

MoHFW developed and implemented an advocacy strategy to promote the campaign and ensure full participation and support from all concerned authorities, including policy-makers from different ministries, top executives of print and electronic media, professional bodies, public leaders, and development partners. This strategy was adopted at the national, divisional, district, City Corporation, local municipality, and *upazila* (sub-district) levels in order to confirm the success of the MRC. As part of this advocacy strategy, the EPI developed materials for information, education and communication (IEC) that were used from the national level to the community level. Additionally, the EPI held advocacy meetings with Bangladesh Medical Association (including with renowned medical professionals), and the Pediatric Association to advise them of the MRC and to seek their support, including for disseminating information to parents. Furthermore, advocacy meetings were held with news editors from the press and electronic media to obtain their support for the publication of articles and messages to help create favorable public awareness and to counter instances of negative publicity, such as the false information that unknown sources circulated through short message service (SMS) to create panic regarding the poor quality of vaccines and the likelihood of causing adverse effects (Fig. 2).

Difficulties occurred around the timing of advocacy events, especially in hard-to-reach and remote areas. In response to these challenges health workers used a variety of communication channels to disseminate messages. For example, a health worker with experience in hard-to-reach areas said:

*We used mosques to make announcements regarding campaign activities in hard-to-reach areas, and if mosques were unavailable, we communicated with the community leaders in order to disseminate our information. Sometimes, we hired volunteers who lived in those areas to help us. We met at the union offices in order to carry out successful work.*

Interviews with children’s caregivers also highlighted that word-of-mouth and community announcements through mosques and the involvement of Health Assistants helped raise awareness in communities about the MRC. As a result, about 81% (95% CI 77.6–84.0) of the mothers expressed satisfaction with the campaign (Table 2) and about 78% (95% CI 74.3–81.1) reported that their children were vaccinated through the campaign (Table 2).

## Discussion

The MRC was highly successful, achieving a 90% coverage rate and increasing awareness about MR vaccine in the communities [15]. The MRC did not disrupt the routine EPI services in the country – a significant accomplishment considering that previous introductions of new vaccines through mass campaigns in other settings have had negative impacts on routine immunization [24]. Successful campaigns depend on the completion of a number of key activities, including planning, budgeting, training, supervision, and monitoring [25]. Our evaluation determined that several factors contributed to the success of the MRC, including political commitment on the part of the MoHFW, effective design of service delivery, a committed health workforce, successful demand generation, and the adaptive management capacity of the EPI and its partners in addressing numerous challenges.

Political commitment for the MRC came from the highest levels of governance, as evidenced by the initiative taken by the Minister of the MoHFW in adjusting the MRC budget and releasing funds, which enabled minimum interruption campaign launch. In other contexts, political commitment to improve health services for women and children has been a critical factor in the success of immunization programs [26, 27]. Similar to other vaccination campaigns in Bangladesh, using both institutional- and community- level service delivery helped to achieve intended outcomes; for example, covering the drop-outs and community children in regular outreach session [13].

Effective design of the campaign and rapid decision-making at different levels of the EPI programme to adjust MRC implementation challenges also contributed to successful implementation of the campaign. The EPI was able to achieve the MRC implementation during a period of political turbulence, by managing the supply of vaccines and related logistics at the community level. Moreover, long-standing partnerships with institutions at the national, district and sub-district level, bolstered EPI’s management capacity during planning and implementation of the MRC. Previous studies have also suggested that support from partner organizations can help boost campaigns, generate demand, manage adverse events following immunization, and strengthen other campaign activities [6, 28]. Our study also indicated that, with the supports of the WHO and UNICEF, the MoHFW managed several challenges including, timely adjustment of vaccine stock-out and logistics supplies. Timely access to accurate information and coordination across multiple levels of the health system enabled rapid identification of implementation problems and ability for EPI and partners to respond quickly with mitigating strategies [26]. The systematic identification of problems by all relevant stakeholders may contribute to a successful vaccination program [28].

Key factors behind successful vaccination campaigns include the level of demand for a vaccine among the targeted population and the ability of service providers to meet it [29]. We observed a dedicated health workforce, with a high level of satisfaction about their involvement in the MRC. Activities designed to improve participation in the campaign helped staff members increase their ability to work under pressure and manage heavy workloads [30, 31]. Moreover, travel allowances, food, and stationery motivated the staff members to perform better [32]. These observations contrast to other those from other studies which reported that using incentives may lead to a culture of dependency among healthcare workers and reduce their motivation to provide routine vaccination and other health services [33].

Previous literature has found that religious beliefs, lack of knowledge, fear of infertility and negative propaganda [22] are the main reasons for people rejecting immunization [34]. Implementation of a comprehensive strategy for demand generation in the target population is crucial to such large campaigns. We observed that advocacy activities at the national to sub-national levels increased the community’s awareness of the MRC and reduced the impact of negative propaganda around the MR vaccine.

In terms of limitations, our evaluation covered only two divisions which may not be enough to provide a comprehensive understanding of the implementation process of the MRC in the entire country. Moreover, some of the findings from this study were derived from a limited number of qualitative interviews which reduced the generalizability of the findings to other settings. Despite these limitations, the strengths of the study included using a range of innovative methods to characterize the implementation process of the MRC, for example, root cause analysis. Additionally, the study provides a holistic understanding of the implementation process of the MRC through a comprehensive triangulation of findings with multiple data sources.

## Conclusions

Overall, successful implementation of the MRC was achieved through a high coverage of the MR vaccine [15] among the children and youth aged 9 months to 14 years, demonstrating that the capacity of the EPI and the partnerships around immunization programs is very strong. We recommend that the EPI programme and its partner institutes work together to strengthen and sustain these partnerships for future vaccine introductions and to respond implementation challenges might occurred at different level of EPI programme, from national to community level. Lessons learnt from vaccine introduction campaigns of other countries are also of value in building capacity for EPI in low-income countries.

## Data Availability

Data that support findings are restricted to authors who have permission from the Gavi Full Evaluation and Research Administration icddr,b, and so are not publicly available. For further information regarding access, please contact Haribondhu Sarma (hsarma@icddrb.org).

## Abbreviations

DGHS: Directorate General of Health Services
EPI: Expanded Program on Immunization
FCE: Full Country Evaluation
IEC: Information, Education and Communication
MoHFW: Ministry of Health and Family Welfare
MR: Measles-Rubella
MRC: Measles-Rubella Campaign
PS: Population-size
SMS: Short Message Service
UNICEF: United Nations Children’s Fund
WHO: World Health Organization
